# Mitochondrial dysfunction and endoplasmic reticulum stress involved in oocyte aging: an analysis using single-cell RNA-sequencing of mouse oocytes

**DOI:** 10.1186/s13048-019-0529-x

**Published:** 2019-06-08

**Authors:** Tao Zhang, Qingsong Xi, Dan Wang, Jingjing Li, Meng Wang, Dan Li, Lixia Zhu, Lei Jin

**Affiliations:** 1 Reproductive Medicine Center, Tongji Hospital, Tongji Medicine College, Huazhong University of Science and Technology, 1095 JieFang Avenue, Wuhan, 430030 People’s Republic of China; 2 Department of Oncology, Tongji Hospital, Tongji Medicine College, Huazhong University of Science and Technology, 1095 JieFang Avenue, Wuhan, 430030 People’s Republic of China; 3 Department of Ophthalmology, Tongji Hospital, Tongji Medicine College, Huazhong University of Science and Technology, 1095 JieFang Avenue, Wuhan, 430030 People’s Republic of China

**Keywords:** Ovarian aging, Single-cell RNA-sequencing, Oocyte, Mitochondria, Complex I, Endoplasmic reticulum stress

## Abstract

**Object:**

To explore the mechanisms of ovarian aging, we performed overall analysis on the age-related alterations of gene expression profiles in mouse germinal vesicle (GV) stage oocytes by means of single-cell RNA-sequencing method (scRNA-seq).

**Methods:**

Two age groups (5-week-old and 32-week-old) female KM mice were used as young and old models. Subsequently, GV oocytes were collected for scRNA-seq. The bioinformatics was performed to analyze and compare the differences of gene expression profile between GV oocytes of young and old mice.

**Results:**

The analysis of scRNA-seq data showed that there were 624 differential expressed genes (DEGs) between two age groups of mouse GV stage oocytes. Four hundred forty-nine DEGs were up-regulated while 175 DEGs were down-regulated in the GV oocytes of the old group. KEGG pathway analysis revealed that the genes involved in mitochondrial function including oxidative phosphorylation and ATP production pathway were significantly down-regulated in GV oocytes of 32-week-old mice, especially the mitochondrial encoded NADH dehydrogenase (mt-Nd), including mt-Nd2, mt-Nd3, mt-Nd4, mt-Nd4L and mt-Nd5. Analysis of DEGs revealed that endoplasmic reticulum stress-related genes including AdipoR2, IRAK-1, RCAN1 and MsrB1 were significantly down-regulated in GV oocytes of 32-week-old mice. Also, analysis of DEGs demonstrated that anti-oxidation-related genes including Erbb3、Rcan1、Gsto2 and Msrb1 were significantly down-regulated in GV oocytes of old group.

**Conclusion:**

The disorder of mitochondrial function, endoplasmic reticulum stress and the reduced antioxidant capability might be involved in the progression of oocyte aging. Especially, the down regulation of mitochondrial encoded subunits of respiratory chain complexes might play critical roles in the relevant mechanisms.

**Electronic supplementary material:**

The online version of this article (10.1186/s13048-019-0529-x) contains supplementary material, which is available to authorized users.

## Introduction

In modern society, more and more females choose to delay reproduction to pursue educational and career goals. As a result, the number of females manifesting with infertility due to ovarian aging increases rapidly as the potentiality of reproduction is limited by ovarian function. The assisted reproductive technology (ART) has undergone a long-term development in recent years, but provides limited help to the pregnancy outcomes of females at advanced maternal age [1–3].

Ovarian aging plays a pivotal role in the decline of female fertility potential, which is characterized as decreasing number of ovarian follicles and age-dependent reduction of oocyte quality [4]. Previous study has reported the comparable clinical pregnancy outcomes between young females using their own oocytes and elder females using oocytes from younger donors in the treatment of in vitro fertilization (IVF), suggesting the key role of oocyte quality reduction in ovarian aging [5].

Despite decades of research, the exact mechanisms of poor oocyte quality due to ovarian aging remain unclear. It has been speculated that increasing aneuploidy rate leads to the poor oocyte and embryo developmental potential in aged females, which results in adverse pregnancy outcomes [6]. Recent investigations have demonstrated that ovarian aging is associated with mitochondrial dysfunction and abnormal structure [7]. The damage of mitochondrial DNA (mtDNA), the disruption of mitochondrial gene expression, and the decline in mitochondrial membrane potential might contribute to mitochondrial dysfunction in the oocytes from aged females [8, 9]. Also, there have been investigations focusing on the age-associated differences in transcriptome of human MII and germ-vesicle (GV) oocyte, demonstrating the possible effects of age on transcriptional activities of oocytes [4, 10]. However, the primary mechanisms of oocyte quality reduction in ovarian aging are still not fully understood.

There have been plenty of researches focusing on the alteration and regulation of gene expression during senescence process in cells, tissues and organs using the global expression profiling skills [11, 12]. However, in the case of oocytes and early embryos, the scarce amounts of biological sample render their transcriptomic analyses until the recent development of single-cell RNA-sequencing (scRNA-seq). With the use of oligo (dT) -based reverse transcription, scRNA-seq was performed on oocytes for the identification of disrupted biological processes and transcripts during senescence [13, 14].

In this study, we choose oocytes from mice of different age groups to control potential confounders due to diverse causes of infertility as well as a large age span, which are quite common in human oocytes used in scientific researches. The global expression profiling of mouse GV oocytes collected from young mice (5-week-old) and old mice (32-week-old) was carried out to investigate the transcriptome differences.

## Materials and methods

### Germinal vesicle (GV) oocyte collection

Six GV oocytes were collected from each of the 6 young KM mice (5-week-old) and 6 old KM mice (32-week-old) (Beijing HFK Bioscience Co. Ltd. China). Briefly, the GV oocytes were collected from mice by ovarian puncture. Then, all remaining cumulus cells were removed by the treatment of hyaluronidase (80 units/ml; Sigma-Aldrich, USA) for 3–5 min in M2 medium (Sigma-Aldrich, USA) and the manipulation of a stripper (Origio, USA) tip. After that, GV oocytes were washed three times in M16 medium (Sigma-Aldrich, USA) for 5 min each and were collected by mouth pipette. All samples were stored at − 80 °C in PicoPure RNA extraction buffer (10 μl; Arcturus Reagents/Molecular Devices, USA).

### Isolation of RNA from the oocytes

Total RNA was extracted and isolated from GV oocytes using Picopure RNA isolation kit (Arcturus Reagents/Molecular Devices, USA) according to the manufacturer’s protocol (Thermo Fisher Scientific, USA). RNA purity was checked using the NanoPhotometer® spectrophotometer (IMPLEN, CA, USA). RNA concentration was measured using Qubit® RNA Assay Kit in Qubit® 2.0 Flurometer (Life Technologies, CA, USA). RNA integrity was assessed using the RNA Nano 6000 Assay Kit of the Bioanalyzer 2100 system (Agilent Technologies, CA, USA).

### Complementary DNA synthesis and amplification, library preparation for transcriptome sequencing

A total amount of 3 μg RNA per sample was used as input material for the RNA sample preparations. Sequencing libraries were generated using NEBNext® Ultra™ RNA Library Prep Kit for Illumina® (NEB, USA) following manufacturer’s recommendations. Index codes were added to attribute sequences to each sample. Briefly, mRNA was purified from total RNA using poly-T oligo-attached magnetic beads. Fragmentation was carried out using divalent cations under elevated temperature in NEBNext First Strand Synthesis Reaction Buffer (5X). First strand cDNA was synthesized using random hexamer primer and M-MuLV Reverse Transcriptase. Second strand cDNA synthesis was subsequently performed using DNA Polymerase I and RNase H. Remaining overhangs were converted into blunt ends via exonuclease/polymerase activities. After adenylation of 3’ends of DNA fragments, NEBNext Adaptor with hairpin loop structure were ligated to prepare for hybridization. In order to select cDNA fragments of preferentially 150~200 bp in length, the library fragments were purified with AMPure XP system (Beckman Coulter, Beverly, USA). Then 3 μl USER Enzyme (NEB, USA) was used with size-selected, adaptor-ligated cDNA at 37 °C for 15 min followed by 5 min at 95 °C before PCR. Then PCR was performed with Phusion High-Fidelity DNA polymerase, Universal PCR primers and Index (X) Primer. At last, PCR products were purified (AMPure XP system) and library quality was assessed on the Agilent Bioanalyzer 2100 system.

### Clustering and sequencing

The clustering of the index-coded samples was performed on a cBot Cluster Generation System using TruSeq PE Cluster Kit v3-cBot-HS (Illumia, USA) according to the manufacturer’s instructions. After cluster generation, the library preparations were sequenced on an Illumina Hi-seq platform and 150 bp paired-end reads were generated.

### RNA-Seq data analysis

The raw reads were processed using CLC Genomics Workbench (CLC, v7.5.1; Qiagen, USA) and the remaining reads of each sample were mapped to the reference genome downloaded from the genome website (http://asia.ensembl.org/Mus_musculus/Location/Genome?ftype=DnaAlignFeature;id=Mm.10). The genome model was built with Bowtie 2, and clean paired-end reads mapped to the reference genome using Tophat 2. The expression values for each gene were calculated as as the expected number of fragments per kilobase of transcript sequence per millions base pairs sequenced (FPKM) by CLC. Differentially expressed genes (DEG) analysis was conducted with DESeq R package (1.18.0). Benjamini and Hochberg’s approach was performed to adjust the resulting *P*-value to control the false discovery rate. Biological function and canonical pathway enrichment analysis (IPA) significance was set at a Benjamini-Hochberg multiple testing correction *P*-value (FDR) < 0.05. When the adjusted P-value < 0.05 and and log2 fold change > 1, the gene would be considered differentially expressed. When the adjusted *P*-value for a gene was < 0.05, the gene would be considered differentially expressed. A heat map was performed for all expressed genes using heatmap3 package in R (v1.1.1). Gene Ontology (GO, http://www.geneontology.org/) enrichment analysis was carried out for all the differentially expressed genes using Ingenuity Pathway Analysis software (IPA; Qiagen, USA). An adjusted P-value≤0.05 was regarded as the indication of significant gene enrichment. Kyoto Encyclopedia of Genes and Genomes (KEGG) analysis of the enrichment of DEGs was performed. An adjusted P-value≤0.05 indicated the significant gene enrichment in pathways.

## Results

### Transcriptome of mouse oocytes

Single-cell RNA-sequencing method was performed to analyze the differences in gene expression between GV oocytes of 5-week-old and 32-week-old mice. For each sample, 12 million total Illumina reads was generated, 80% of which was mapped to the reference genome downloaded from the genome website (http://asia.ensembl.org/Mus_musculus/Location/Genome?ftype=DnaAlignFeature;id=Mm.10) **(**Additional file 1: Table S1**)**. The expression values for each gene were calculated as the expected number of fragments per kilobase of transcript sequence per millions base pairs sequenced (FPKM). Seventeen thousand five hundred eighty-six expressed genes in mouse oocytes were identified on the basis of a cut-off of FPKM > 0.0 **(**Additional file 1: Table S2**)**.

**Fig. 1:**
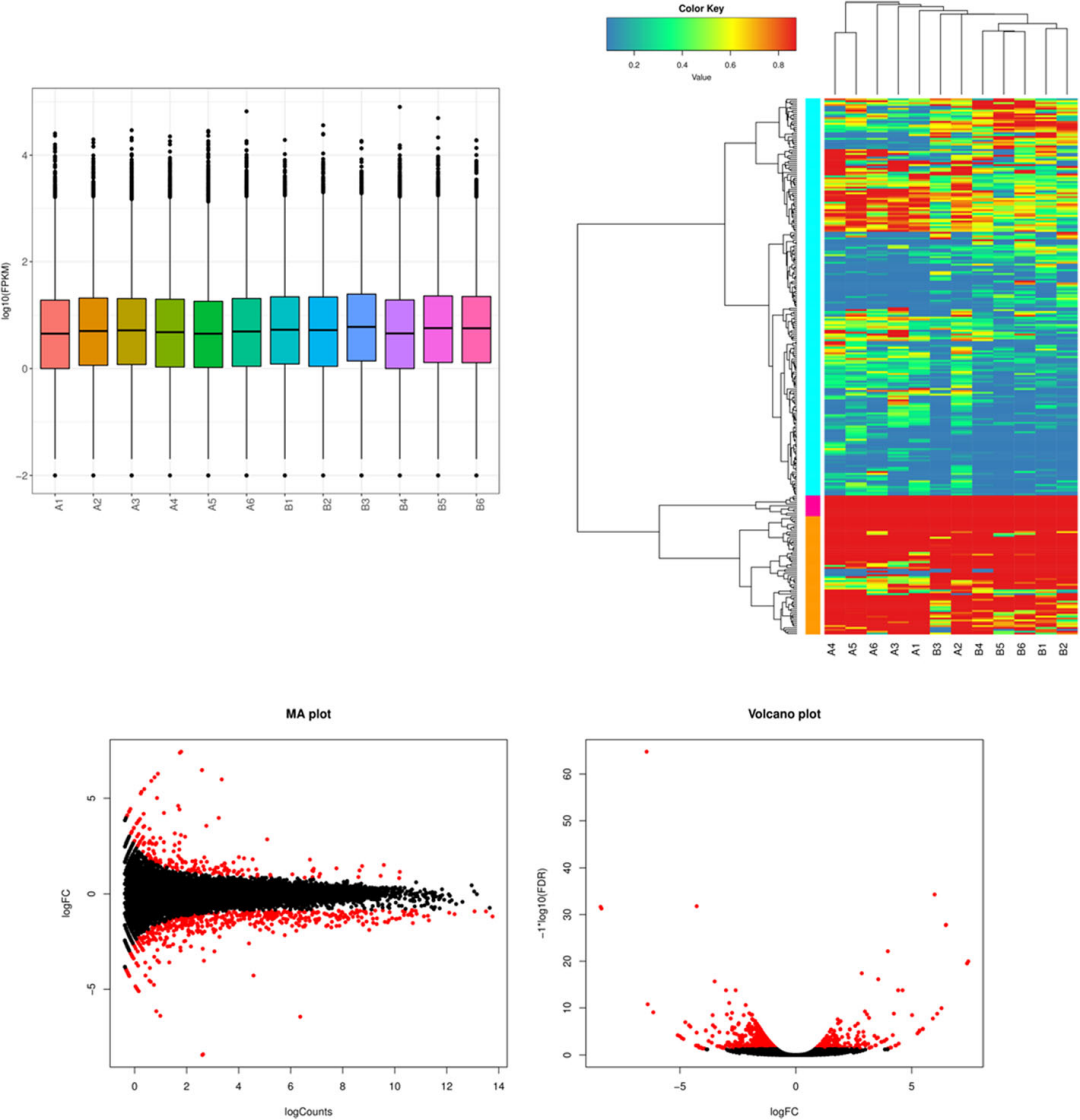
Gene expression levels of GV stage oocytes from old (32-week) mice and young (5-week) mice. **a** The boxplot showed the global gene expression level (log10 FPKM) of each sample. **b** The heat map showed the clustering analysis of gene expression levels (FPKM) in each sample. **c** MA plot and volcano plot showed the differentially expressed genes (DEGs) between oocytes from old (32-week-old) mice and young (5-week-old) mice. A, oocytes from old (32-week-old) mice. B, oocytes from young (5-week-old) mice. The red dots in MA plot and volcano plot refer to DEGs while black dots represent the genes that were not diferently expressed in 5-week-old mice vs 32-week-old mice

Boxplots of log10-transformed FPKM values for each samples revealed that these samples had consistent overall range and distribution of the FPKM values **(**Fig. 1a**)**, indicating the single-cell RNA-sequencing data of this study was of reliability, reproducibility and high quality. Cluster analysis of gene expression levels (FPKM) in each sample showed the differences of gene expression profile between GV oocytes of 5-week-old and 32-week-old mice, which revealed that the RNA-sequencing data of this study met the conditions for differential expression analysis **(**Fig. 1b**)**.

### Global gene expression characteristics of oocyte aging

Significant DEGs was considered as the genes with FPKM > 1 and adjusted *p*-value < 0.05. Generally, 624 genes were differentially expressed between GV oocytes of 5-week-old and 32-week-old mice. Among these DEGs, 449 genes were up-regulated while 175 genes were down-regulated in the GV oocytes of 32-week-old mice compared with those at 5-weeks of age **(**Fig. 1c**)**.

**Fig. 2:**
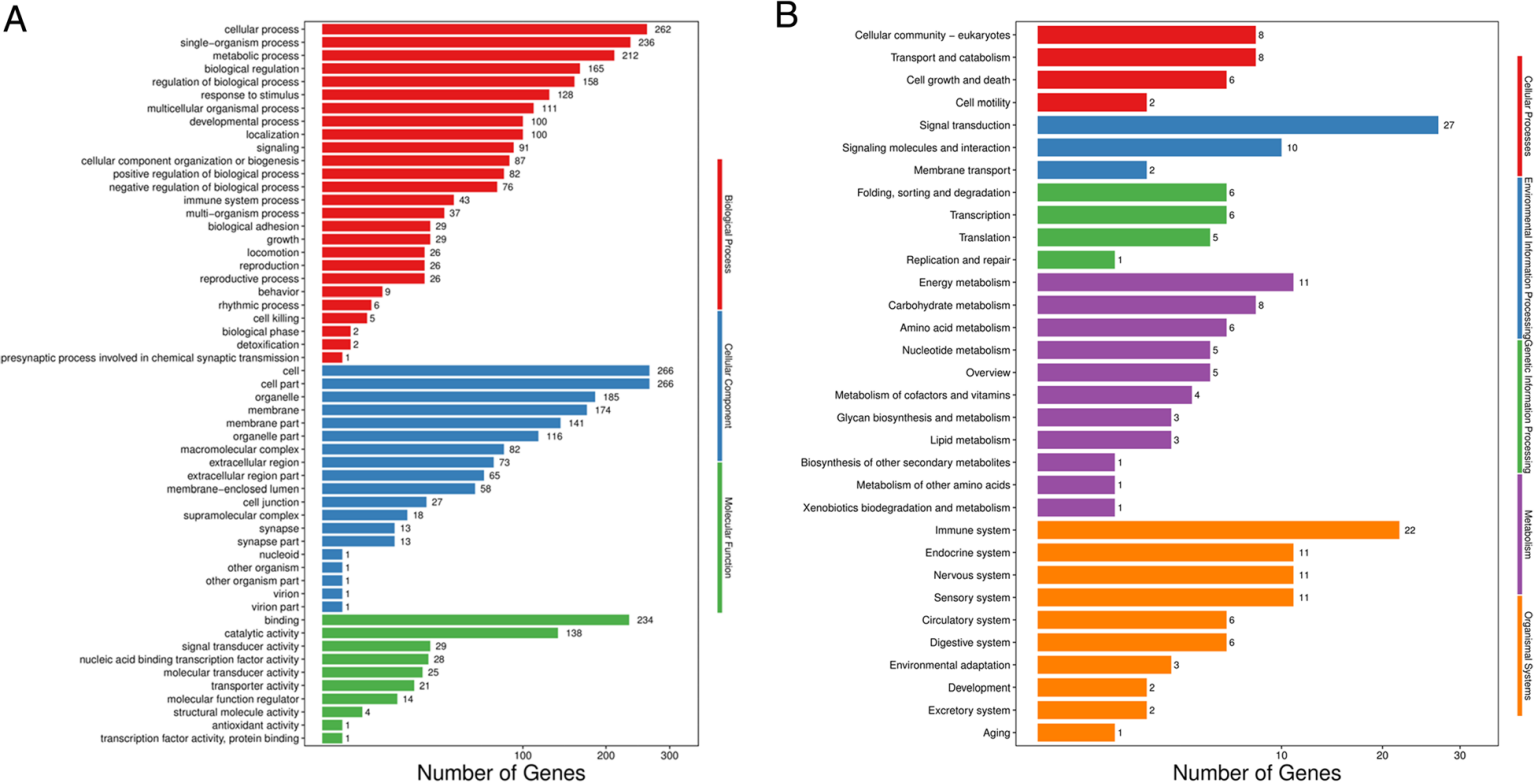
The analysis of differentially expressed genes (DEGs) between GV stage oocytes from old (32-week) mice and young (5-week) mice. **a** GO (Gene Ontology, http://www.geneontology.org/)enrichment analysis on DEGs. Red refers to terms relating to biological processes, blue refers to terms relating to cellular components and green refers to terms relating to molecular function. **b** KEGG (Kyoto Encyclopedia of Genes and Genomes) pathways enriched from DEGs. Red refers to terms relating to cellular processes, blue refers to terms relating to environmental information processing and green refers to terms relating to genetic information processing, purple represents terms relating to metabolism and orange represents terms relating to organismal systems

GO enrichment analysis was conducted to identify the distribution of age-related differential gene expression in biological functions **(**Fig. 2a). “Cellular process” and “single-organism process” were the most significant enrichment terms in cellular components category, including 262 and 236 DEGs, respectively. “Cell” and “cell part” were the most significant enrichment terms in biological processes category, including 266 and 266 DEGs, respectively. “Binding” and “catalytic activity” were the most significant enrichment terms in biological processes category, including 234 and 138 DEGs, respectively.

KEGG enrichment analysis was performed to identify the distribution of age-related differential gene expression in biological pathways **(**Fig. 2b**)**. “Cellular community-eukaryotes” was the most significant enrichment terms in cellular process category, including 8 DEGs. “Signal transduction” was the most significant enrichment terms in environmental information processing category, including 27 DEGs. “Folding, sorting and degradation” was the most significant enrichment terms in genetic information processing category, including 6 DEGs. “Energy metabolism” was the significant most enrichment terms in metabolism category, including 11 DEGs. “Immune system” was the most significant enrichment terms in organismal systems category, including 22 DEGs.

### Age-related differential gene expression in mitochondrial function

**Fig. 3:**
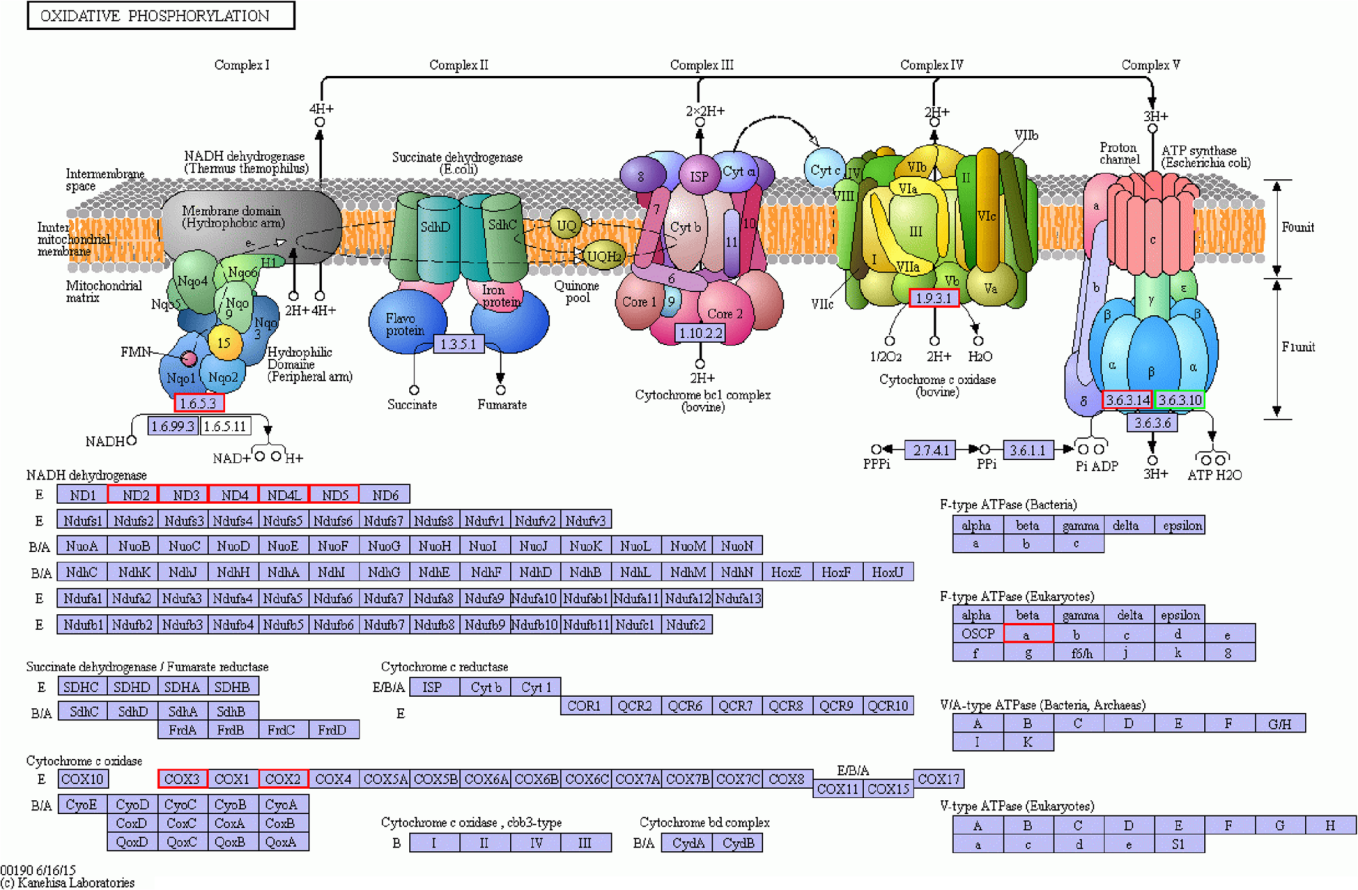
The KEGG pathway of oxidative phosphorylation pathway responds to oocyte aging, and the genes highlighted in red are enriched and significantly down-regulated in oocytes from the old mice group. Most of these DEGs were encoded in the mitochondrial genome and participated in mitochondrial respiratory chain by encoding mitochondrially encoded nicotine-amide adenine dinucleotide (NADH) dehydrogenase (mt-Nd), the key protein subunits of Complex I. Those DEGs included mt-Nd2, mt-Nd3, mt-Nd4, mt-Nd4L and mt-Nd5. Also, the genes encoding the subunits Complex IV and Complex V of respiratory chain including mitochondrially encoded ATP synthase 6 (mt-ATP6), cytochrome c oxidase subunit II (COX2) and mitochondrial Cytochrome c oxidase subunit III (COX3) were found significant down-regulated in the GV oocytes of 32-week-old mice

KEGG enrichment analysis revealed that most DEGs involved in oxidative phosphorylation of energy metabolism pathway was down-regulated in the GV oocytes of 32-week-old mice compared with those at 5-weeks of age (Table 1). Especially, most of these DEGs were encoded in the mitochondrial genome and participated in mitochondrial respiratory chain by encoding mitochondrially encoded nicotine-amide adenine dinucleotide (NADH) dehydrogenase (mt-Nd), the key protein subunits of complex I. Those DEGs included mt-Nd2, mt-Nd3, mt-Nd4, mt-Nd4L and mt-Nd5 **(**Table 1) (Fig. 3). Also, the genes encoding the subunits complex IV and complex V of respiratory chain including mitochondrially encoded ATP synthase 6 (MT-ATP6), cytochrome c oxidase subunit II (COX2) and mitochondrial Cytochrome c oxidase subunit III (COX3) were found significantly down-regulated in the GV oocytes of 32-week-old mice.

**Table 1:** GV stage oocyte expression of differentially expressed genes (DEG) related to oxidative phosphorylation, endoplasmic reticulum stress and oxidative stress between old (32-week) mice and young (5-week) mice. GV, germinal vesicle. FPKM, the expression of each transcript in each sample was calculated as the expected number of fragments per kilobase of transcript sequence per millions base pairs sequenced. The threshold of DEGs was the genes with a FDR < 0.05 and log_2_FC > 1 or log_2_FC < − 1

Gene	32w GV FPKM	5w GV FPKM	log2(fold change)	adjusted *p*-value	significant
Oxidative phosphorylation					
mt-Nd1	469.13	879.60	0.91	5.07E-03	no
mt-Nd2	107.87	274.79	1.35	5.07E-03	yes
mt-Nd3	123.74	539.20	1.87	1.23E-07	yes
mt-Nd4	129.35	326.45	1.34	5.07E-03	yes
mt-Nd4l	178.38	537.35	1.39	4.18E-04	yes
mt-Nd5	56.33	129.78	1.20	5.07E-03	yes
mt-Nd6	38.15	87.56	1.20	6.72E-01	no
mt-Atp6	1944.10	4816.67	1.16	1.01E-03	yes
COX2	606.11	1857.79	1.46	1.06E-05	yes
COX3	823.32	2605.95	1.52	4.09E-06	yes
Endoplasmic reticulum stress					
Rcan1	3.30	7.34	1.15	5.07E-03	yes
Msrb1	8.20	18.55	1.18	2.70E-02	yes
Adipor2	4.89	8.65	1.22	1.39E-02	yes
Irak1	2.58	4.71	1.11	3.10E-02	yes
Xaf1	1.00	3.36	1.75	5.07E-03	yes
Oxidative stress					
Erbb3	0.94	2.93	1.64	5.07E-03	yes
Rcan1	3.30	7.34	1.15	5.07E-03	yes
Gsto2	80.08	143.83	1.12	2.47E-02	yes
Msrb1	8.20	18.55	1.18	2.70E-02	yes
ACAA1	0.67	1.35	2.74	8.94E-03	yes

### Age-related differential gene expression in endoplasmic reticulum stress

The analysis of DEGs revealed that a group of genes participating in the protection against endoplasmic reticulum stress were down-regulated in the GV oocytes of 32-weeks mice, including adiponectin receptor 2 (AdipoR2), interleukin 1 receptor associated kinase 1 (IRAK-1), regulator of calcineurin 1 (Rcan1) and methionine sulfoxide reductase B1 (MsrB1) **(**Table 1**)**.

### Age-related differential gene expression in oxidative stress

A group of DEGs participating in the protection against oxidative stress were also down-regulated in the GV oocytes of 32-weeks mice. KEGG enrichment analysis revealed that acetyl-CoA acyltransferase 1 (ACAA1), involved in the metabolic pathway of peroxisome, was significantly down-regulated in the GV oocytes of 32-weeks mice. Besides, the genes related to antioxidation was significantly down-regulated in the GV stage oocytes of 32-weeks mice, including erb-b2 receptor tyrosine kinase 3 (Erbb3), regulator of calcineurin 1 (Rcan1), glutathione S-transferase omega 2 (Gsto2) and methionine sulfoxide reductase B1 (MsrB1) (Table 1).

## Discussion

The exact mechanisms underlying the ovarian aging are still not well understood. Senescence of oocyte is believed to play a central role in ovarian aging. Single-cell RNA-sequencing method provides the access to the detection and quantification of the entire transcripts in a single cell, accelerating greatly the investigations on gene expression profile of single oocyte. Previous studies have compared the differences of gene expression profile in GV or MII stage oocytes from young and old individuals [4, 10], which were limited by the suboptimal quality of the oocytes as most of them were retrieved from infertility females. Moreover, the broad age range could act as another important confounding factor. Thus in the current study, to circumvent these limitations, single-cell RNA-sequencing method was conducted on GV stage mouse oocytes from young group of 5-weeks and old group of 32-weeks for the identifications of age-related gene expression profile changes in oocytes.

Generally, our data revealed that oocytes from young and old group had similar gene expression profile at the transcriptome level. There were only 624 genes (3.5%) exhibiting statistically significant expression differences, among which 449 genes were up-regulated while 175 genes were down-regulated in the GV stage oocytes of old group. The number of DEGs was similar to previous investigators [11]. However, the differential gene expression analysis revealed that many DEGs were related to important cellular function. These DEGs were enriched for biological functions and metabolic pathway associated with cellular growth.

Our study demonstrated that the disorder of mitochondrial function including oxidative phosphorylation and ATP production pathway was involved in the progression of oocyte aging. Mitochondrion is an important organelle in the eukaryotic cells, which acts as a major player in energy metabolism and ATP production [15]. Therefore, the dysfunction of mitochondria could lead to decline of ATP production. It has been speculated that the higher ATP content in human and mouse MII oocytes was associated with better embryo potential for development and implantation [16]. The normal function of mitochondria is the guarantee of oocyte quality and embryo developmental potentiality, while oocyte aging is related to mitochondrial dysfunction and disturbance of energy metabolism [17–19].

Particularly, the down regulation of mitochondrially encoded subunits of respiratory chain complexes testified in our study might play key roles in the age-related dysfunction of mitochondria. The complex I, or nicotine-amide adenine dinucleotide (NADH) dehydrogenase, is the largest protein complex in mitochondrial respiratory chain [20–22]. Complex I consists of more than 40 subunits, seven of which are encoded by mtDNA, named as mitochondrially encoded NADH dehydrogenase (mt-Nd), including mt-Nd1, mt-Nd2, mt-Nd3, mt-Nd4, mt-Nd4L, mt-Nd5 and mt-Nd6. Additionally, there are 14 corresponding homologous genes in bacteria for the seven mtDNA-encoded genes, indicating the importance of these subunits for cellular survival [23]. Besides, complex I also served as the main entrance for electrons to the respiratory chain. Simple complex I defect is the most common genetic disease in the dysfunction of mitochondrial respiratory chain, causing a diverse range of clinical symptoms including lactic acidosis, cardiomyopathy, leukoencephalopathy and muscle atrophy [24]. The functional test of mitochondrial respiratory chain in the muscle of these patients showed reduced rate of ATP production in mitochondria [25]. In general, the dysfunction of mt-Nd genes might lead to the disorder of mitochondrial respiratory chain and the decline of ATP production.

Our study also indicated that the endoplasmic reticulum might play essential roles in the deterioration of oocyte quality in oocyte aging [26–28]. These DEGs included AdipoR2, IRAK-1, RCAN1 and MsrB1. Studies have focused on the disorder of Ca2+ oscillations and homeostasis due to the dysfunction of endoplasmic reticulum in aged oocytes [26, 27, 29]. There are also researches reporting the possible relationship between reactive oxygen species (ROS) -induced mitochondrial damage and the disorder of endoplasmic reticulum during oocyte aging progression [27]. The analysis of DEGs in this study demonstrated that some genes associated with the regulation of endoplasmic reticulum stress (ER stress) were down-regulated in GV stage oocytes from old group, indicating the possible roles of ER stress in oocyte aging.

ROS is active chemically and participates in redox signaling to regulate cellular processes [30–32]. However, ROS can attack protein, lipids and DNA, causing damage to cellular structure and function. Under the physiological conditions, mitochondrial respiratory chain is the main source of ROS and there is a balance between ROS production and ROS scavenging systems in cell [31]. In the current research, the analysis of DEGs revealed that the anti-oxidative stress related genes were significantly down-regulated in the oocytes of old mice, including Erbb3, Rcan1, Gsto2 and Msrb1. Our data indicated that the antioxidant capacity of oocytes from old mice is attenuated, which might lead to the accumulation of ROS and disorder of cellular process. The high level of ROS in human follicular fluid was related to poorer developmental potentiality of oocytes [33], while adding antioxidants to in vitro culture environment of porcine oocytes improved the oocyte quality and blastocyst formation rate dramatically [34].

In this study, we found the genes involved in oxidative phosphorylation pathway were significant down-regulated in the GV stage oocytes from 32-week-old mice compared with those from 5-week-old ones. Especially, most of these DEGs were located in mtDNA and participated in the encoding of mitochondrially encoded NADH dehydrogenase, the key protein subunits of complex I, including mt-Nd2, mt-Nd3, mt-Nd4, mt-Nd4L and mt-Nd5. Our results indicated that the function of oxidative phosphorylation and mitochondrial respiratory chain would be disturbed in the progression of oocyte aging. Interestingly, the mitochondrially encoded subunits involved in respiratory chain might be affected more significantly and earlier, as mitochondrial DNA lack the protection of histones and effective DNA repair system [35, 36]. Besides, working as the main source of ROS, mitochondrial DNA will face more attack from oxidative stress [35, 36]. As the main entrance for electrons to the respiratory chain, the dysfunction of complex I will lead to the disorder of mitochondrial respiratory chain and decreasing ATP production, causing variable clinical manifestations [24, 25, 37]. Our analysis indicated that aging would adversely affect mitochondrial respiratory chain in oocytes, especially the mitochondrially encoded subunits in complex I.

The limiting aspect of our study was the number of oocytes analyzed. In addition, the transcriptome of mice is still different from that of human. A well-designed investigation with large sample size will be needed to test our hypothesis even further.

To be concluded, with the application of scRNA-seq, this study revealed that the dysfunction of mitochondrial respiratory chain, endoplasmic reticulum stress and reduced antioxidant capacity might all be involved in the progression of oocyte aging. Especially, the disorder of mitochondrial DNA-encoded subunits of respiratory chain complexes might play a central role in this process.

## Additional file

Additional file 1: **Table S1.** The mapping of sequencing reads of samples to reference genomes the reference genome downloaded from the genome website (http://asia.ensembl.org/Mus_musculus/Location/Genome?ftype=DnaAlignFeature;id=Mm.10). 32w,the GV oocytes from 32-weeks mice 5w, the GV oocytes from 5-weeks mice. **Table S2.** Analysis of gene expression in samples at different levels of expression FPKM, the expression of each transcript in each sample was measured as the expected number of fragments per kilobase of transcript sequence per millions base pairs sequenced 32w, the GV oocytes from 32-weeks mice 5w, the GV oocytes from 5-weeks mice. (DOC 50 kb)

## Data Availability

The reference genome of mice was downloaded from the genome website (http://asia.ensembl.org/Mus_musculus/Location/Genome?ftype=DnaAlignFeature;id=Mm.10). Sequencing data will be uploaded to pubmed if this manuscript was accepted.

## Abbreviations

ACAA1: Acetyl-CoA acyltransferase 1
AdipoR2: Adiponectin receptor 2
ART: Assisted reproductive technology
COX2: Cytochrome c oxidase subunit II
COX3: Cytochrome c oxidase subunit III
DEG: Differentially expressed genes
ER: Endoplasmic reticulum
Erbb3: erb-b2 receptor tyrosine kinase 3
ESRD: End-stage renal disease
FPKM: Expected number of fragments per kilobase of transcript sequence per millions base pairs sequenced
GO: Gene Ontology
Gsto2: Glutathione S-transferase omega 2
GV: Germinal vesicle
IRAK-1: Interleukin 1 receptor associated kinase 1
IVF: In vitro fertilization
KEGG: Kyoto Encyclopedia of Genes and Genomes
MsrB1: Methionine sulfoxide reductase B1
MT-ATP6: Mitochondrially encoded ATP synthase
mtDNA: Mitochondrial DNA
mt-Nd: Mitochondrial encoded NADH dehydrogenase;
NADH: Nicotine-amide adenine dinucleotide
Rcan1: Regulator of calcineurin 1
ROS: Reactive oxygen species
scRNA-seq: Single-cell RNA-sequencing method
SelR: Selenoprotein R
