# Supplementary material for: Mitochondrial dysfunction and endoplasmic reticulum stress involved in oocyte aging: an analysis using single-cell RNA-sequencing of mouse oocytes
Source: J Ovarian Res. 2019 Jun 8;12:53. doi: 10.1186/s13048-019-0529-x (PMC6556043; doi:10.1186/s13048-019-0529-x)
Supplement: Supplementary file 1 — Table S1. The mapping of sequencing reads of samples to reference genomes the reference genome downloaded from the genome website (http://asia.ensembl.org/Mus_musculus/Location/Genome?ftype=DnaAlignFeature;id=Mm.10). 32w,the GV oocytes from 32-weeks mice 5w, the GV oocytes from 5-weeks mice. Table S2. Analysis of gene expression in samples at different levels of expression FPKM, the expression of each transcript in each sample was measured as the expected number of fragments per kilobase of transcript sequence per millions base pairs sequenced 32w, the GV oocytes from 32-weeks mice 5w, the GV oocytes from 5-weeks mice. (DOC 50 kb) [file 13048_2019_529_MOESM1_ESM.doc]

Supplementary Material

| **Sample** | **Total Reads** | **Total mapping rate** | **Left reads mapped** | **Left reads multiple mapped** | **Right reads mapped** | **Right reads multiple mapped** |
| --- | --- | --- | --- | --- | --- | --- |
| 32w-1 | 13,977,408 | 11,006,038(78.7%) | 5,481,203(78.4%) | 1,176,277(21.5%) | 5,524,835(79.1%) | 1,156,196(20.9%) |
| 32w-2 | 12,401,694 | 9,498,573(76.6%) | 4,752,850(76.6%) | 774,165(16.3%) | 4,745,723(76.5%) | 754,849(15.9%) |
| 32w-3 | 10,457,474 | 8,145,498(77.9%) | 4,100,809(78.4%) | 840,236(20.5%) | 4,044,689(77.4%) | 811,256(20.1%) |
| 32w-4 | 12,894,862 | 10,415,388(80.8%) | 5,231,983(81.1%) | 1,016,958(19.4%) | 5,183,405(80.4%) | 989,250(19.1%) |
| 32w-5 | 11,620,600 | 9,299,439(80.0%) | 4,697,383(80.8%) | 1,200,416(25.6%) | 4,602,056(79.2%) | 1,155,529(25.1%) |
| 32w-6 | 11,343,674 | 8,895,602(78.4%) | 4,475,789(78.9%) | 879,951(19.7%) | 4,419,813(77.9%) | 850,646(19.2%) |
| 5w-1 | 12,757,466 | 9,754,440(76.5%) | 4,855,546(76.1%) | 616,021(12.7%) | 4,898,894(76.8%) | 612,638(12.5%) |
| 5w-2 | 14,022,168 | 9,890,085(70.5%) | 4,850,682(69.2%) | 666,159(13.7%) | 5,039,403(71.9%) | 675,774(13.4%) |
| 5w-3 | 9,499,236 | 7,534,646(79.3%) | 3,831,581(80.7%) | 504,767(13.2%) | 3,703,065(78.0%) | 486,619(13.1%) |
| 5w-4 | 13,359,556 | 10,546,848(78.9%) | 5,296,586(79.3%) | 994,274(18.8%) | 5,250,262(78.6%) | 967,696(18.4%) |
| 5w-5 | 10,073,286 | 7,633,206(75.8%) | 3,810,779(75.7%) | 582,869(15.3%) | 3,822,427(75.9%) | 571,806(15.0%) |
| 5w-6 | 12,246,330 | 9,219,372(75.3%) | 4,600,112(75.1%) | 689,948(15.0%) | 4,619,260(75.4%) | 678,312(14.7%) |

**Supplementary Table S1. The mapping of sequencing reads of samples to reference genomes**. the reference genome downloaded from the genome website(http://asia.ensembl.org/Mus_musculus/Location/Genome?ftype=DnaAlignFeature;id=Mm.10). 32w,the GV oocytes from 32-weeks mice. 5w, the GV oocytes from 5-weeks mice.

| **Sample** | **Expressed_Gene** | **Total_Gene** | **0(FPKM)** | **0~1(FPKM)** | **1~3(FPKM)** | **3~15(FPKM)** |
| --- | --- | --- | --- | --- | --- | --- |
| 32w-1 | 17,777 | 30,956 | 13,179(42.57%) | 4,455(14.39%) | 3,114(10.06%) | 5,084(16.42%) |
| 32w-2 | 17,704 | 30,956 | 13,252(42.81%) | 4,116(13.30%) | 3,087(9.97%) | 5,081(16.41%) |
| 32w-3 | 17,445 | 30,956 | 13,511(43.65%) | 3,953(12.77%) | 3,123(10.09%) | 5,124(16.55%) |
| 32w-4 | 17,960 | 30,956 | 12,996(41.98%) | 4,347(14.04%) | 3,047(9.84%) | 5,241(16.93%) |
| 32w-5 | 17,723 | 30,956 | 13,233(42.75%) | 4,328(13.98%) | 3,252(10.51%) | 5,129(16.57%) |
| 32w-6 | 17,544 | 30,956 | 13,412(43.33%) | 4,193(13.55%) | 2,932(9.47%) | 5,115(16.52%) |
| 5w-1 | 17,655 | 30,956 | 13,301(42.97%) | 3,946(12.75%) | 3,059(9.88%) | 5,106(16.49%) |
| 5w-2 | 17,799 | 30,956 | 13,157(42.50%) | 4,243(13.71%) | 2,923(9.44%) | 5,031(16.25%) |
| 5w-3 | 16,527 | 30,956 | 14,429(46.61%) | 3,354(10.83%) | 2,834(9.15%) | 4,817(15.56%) |
| 5w-4 | 17,763 | 30,956 | 13,193(42.62%) | 4,452(14.38%) | 3,080(9.95%) | 5,088(16.44%) |
| 5w-5 | 17,320 | 30,956 | 13,636(44.05%) | 3,737(12.07%) | 2,879(9.30%) | 5,130(16.57%) |
| 5w-6 | 17,809 | 30,956 | 13,147(42.47%) | 3,895(12.58%) | 2,896(9.36%) | 5,358(17.31%) |

**Supplementary Table S2. Analysis of gene expression in samples at different levels of expression**. FPKM, the expression of each transcript in each sample was measured as the expected number of fragments per kilobase of transcript sequence per millions base pairs sequenced. 32w,

the GV oocytes from 32-weeks mice. 5w, the GV oocytes from 5-weeks mice.
